# Development of Novel Symptom Score to Assist in Screening for Exocrine Pancreatic Insufficiency

**DOI:** 10.3390/epidemiologia6030048

**Published:** 2025-08-12

**Authors:** Dana M. Lewis, Amanda Landers

**Affiliations:** 1 OpenAPS, Seattle, WA 98101, USA; 2 Department of Medicine, University of Otago, Christchurch 8011, New Zealand; amanda.landers@otago.ac.nz

**Keywords:** EPI, PEI, pancreatic enzyme replacement therapy, PERT, pancreas, symptoms, quality of life, QOL, outcomes, diagnosis, screening

## Abstract

Background: Exocrine pancreatic insufficiency (EPI or PEI) often goes undiagnosed or misdiagnosed due to nonspecific symptomatology. Clinicians may focus primarily on symptoms like diarrhea and steatorrhea, potentially overlooking more prevalent symptoms. Methods: Our research describes the development and evaluation of the Exocrine Pancreatic Insufficiency Symptom Score (EPI/PEI-SS), a novel patient-generated symptom score designed to capture a wide range of EPI-related symptoms and quantify symptoms based on frequency and severity (score ranges 0–225). This preliminary real-world study assessed the efficacy of the EPI/PEI-SS in differentiating between individuals with and without EPI. To examine disparities between participants with and without EPI, average symptom frequency, severity, and overall score relationships were assessed, as well as sub-analyses based on other health co-conditions. Results: In total, 324 participants (155 with EPI and 169 without) completed the EPI/PEI-SS online. Individuals with EPI reported significantly higher EPI/PEI-SS scores (98.11, range 1–213) indicating a greater symptom burden compared with those without EPI (38.86, range 0–163). Conclusions: The EPI/PEI-SS appears to effectively differentiate between EPI and non-EPI participants, including non-EPI participants with other GI conditions. The EPI/PEI-SS demonstrates the potential to identify EPI and distinguish symptoms of EPI from other GI conditions, as evaluated with frequency and severity. Future research could replicate the study alongside fecal elastase testing, to determine whether it can be used additionally or alternatively for EPI diagnosis.

## 1. Introduction

Exocrine pancreatic insufficiency (EPI or PEI), despite its considerable impact on quality of life, often goes undiagnosed [1] or misdiagnosed [2] due to the nonspecific and variable nature of its symptoms, such as excessive gas, abdominal pain, messy or smelly stools, or diarrhea [3]. EPI is a disease of pancreatic exocrine dysfunction and impaired digestive function [4], occurring from a variety of pancreatic- and non-pancreatic-related disorders [5] and commonly in a variety of conditions ranging from cystic fibrosis [6] and chronic pancreatitis [7] to more prevalent conditions such as diabetes [8,9], as well as in the general population [10]. The treatment for EPI is pancreatic enzyme replacement therapy (PERT), which has been shown to be safe, well-tolerated, and effective therapy for EPI [11].

Diagnosing EPI is challenging due to the lack of reliable tools [12], including fecal elastase testing, whose variable sensitivity and specificity limit diagnostic usefulness for EPI [13]. Other tests such as the 72 h fecal fat collection are burdensome and disliked by patients and clinicians [14]. Evidence illustrates the likelihood that the previous perceived “clear” cutoff of fecal elastase levels may exclude people who would benefit from PERT [15]. Additional tools which improve the diagnosis of EPI, including the development of non-invasive symptom evaluation tools, may increase access to treatment, and ultimately improve the quality of life of people with EPI globally.

There is little evidence in the literature of patient-led development of diagnostic and screening tools related to EPI. One current symptom tool for EPI was developed for individuals with EPI and only either cystic fibrosis or chronic pancreatitis [16]. Because these conditions (CF [17] and CP [18]) themselves are rare overall, especially when compared with the estimated general population prevalence of EPI itself [10], individuals with these conditions are likely a small fraction of the EPI population. In evaluation of this symptom tool for people with diabetes and EPI, it was observed to not be a reliable, objective scoring system for identifying EPI [19], nor for those with bariatric surgery [20]. Subsequent tools evaluating symptom burden have also focused only on specific populations impacted by EPI, even specifically excluding other common co-conditions associated with EPI [21], and these tools have not been validated in a more generalized population of people with EPI. Thus, there remains a lack of a symptom tool for people with EPI which has been validated in a representative population and which can serve people living with EPI globally.

In this study we describe the development of a patient-developed symptom scoring tool called the EPI/PEI-SS (Exocrine Pancreatic Insufficiency Symptom Score), and a preliminary evaluation with real-world pilot data of the EPI/PEI-SS in both individuals living with EPI and individuals from a generalized population who do not identify as having EPI.

## 2. Materials and Methods

### 2.1. Ethics

This was a low-risk community-developed and -led study creating a symptom scoring tool by patients, for patients. General best practices of ethical research were followed. These matched the design and implementation of previously published online surveys with, and by, this community [22]. Participants of the survey were informed about the purpose and design of the survey. Participants were also informed that no identifying information would be collected and that their anonymous responses would be used for initial validation of the symptom scoring tool and be shared publicly in an unidentifiable way. Participants were aware of who designed the survey (DL) and informed that they had no obligations to fill out the survey and could stop at any time. This anonymous, minimal-risk survey research met the criteria for exemption under the U.S. Common Rule (§46.104 d (2)) [23] and the HIPAA Privacy Rule (§164.514 b) [24].

### 2.2. Symptom Score Development

To address the challenges of diagnosis and screening for EPI, a new and comprehensive symptom scoring tool was developed for evaluating the frequency and severity of common EPI symptoms. The symptom score (EPI/PEI-SS) was developed through a review of the existing literature [10,25] (lists of presenting symptoms, differences noted across sub-populations within EPI, evidence from a systematic review on PERT efficacy [25] regarding symptoms) combined with patient-generated lists of EPI symptoms. Previous EPI symptom tools were also evaluated [16,21]. Symptoms were refined and grouped into three key categories (Table 1) and scored by multiplying the frequency (0–5 possible) and severity (0–3 possible) for each symptom (0–15 total). The 15 symptoms were then added for a total possible score for EPI/PEI-SS of 225.

### 2.3. Initial Symptom List Validation

To determine whether the calculation of frequency and severity could distinguish EPI-related symptoms from normal, non-pathological gastrointestinal symptoms (such as occasional bloating, gas, or diarrhea), an initial pilot survey was completed (Google Forms, Alphabet) by eleven individuals with and without known EPI. All participants of the survey provided informed consent. Upon pilot survey completion, scores were calculated for each sub-group and the overall score was generated. For each symptom category, central tendencies and variability were analyzed, and Pearson correlation coefficients were computed between the subtotals of each of the three categories. Feedback reported from any symptoms not covered by the survey was analyzed. As the pilot results confirmed likely ability to distinguish between non-pathological gastrointestinal symptoms in those without known GI conditions, the EPI/PEI-SS was amended for further evaluation.

### 2.4. Data Collection

Following the initial pilot study, the symptom scoring tool survey was posted to various social media platforms such as Twitter, Facebook, and LinkedIn, as well as specific Facebook groups such as “Living With Exocrine Pancreatic Insufficiency Support Group” and “CGMITC Off Topic” (a diabetes-related group). Input, approval, and permission from group administrators was requested prior to posting. In the survey (Google Forms, Alphabet), non-identifiable demographic categories were collected, including other health conditions. Those who identified as having EPI were asked about the duration of their condition; whether they had fecal elastase testing and if so, the result; whether they were taking pancreatic enzyme replacement therapy (PERT) and if so, the type (prescription or not) and dose size; and a self-rated assessment of their dose (whether it was the appropriate amount to manage their EPI symptoms). All participants (with/without EPI) then identified the frequency and severity, if any, of the 15 symptoms. There was a final, open-ended question to identify any other gastrointestinal symptoms they experience that was not reflected within the survey.

### 2.5. Data Analysis

Descriptive and summary statistics were used to analyze demographic distributions and calculate survey sub-scores and total scores, incorporating frequency and severity mapping for each symptom. Analysis was conducted for the total cohort and stratified by EPI and non-EPI groups to identify differences, including the top and bottom three symptoms per group. Lasso regression and correlation matrices elucidated symptom relationships and contributions to total scores.

To examine disparities between participants with and without EPI, average symptom frequency, severity, and scoring relationships were assessed. Given the non-parametric nature of the data, Mann–Whitney U tests were used for comparisons, supplemented by Cohen’s d for effect size and Cronbach’s alpha for reliability. Predictors for sub-scores were ranked and ANOVA, *t*-tests, and Tukey’s HSD (honestly significant difference) were applied to explore data distributions and variances.

A conditions analysis evaluated the contributions of other conditions to total score, including for those without EPI yet with other GI conditions (defined as any one of IBS, lactose intolerance, GERD, other food intolerances, gastroparesis (delayed gastric emptying), or celiac disease), as well as an analysis of type 1 diabetes status. Given the high number of participants with type 1 diabetes, related to an author’s (DL) online network and prevalence of EPI among people with diabetes [8], additional sub-group analysis was performed to confirm no practical nor statistically significant differences influencing the symptom scores regardless of status of type 1 or type 2 diabetes (reported in a subsequent article [26]).

## 3. Results

A total of 324 surveys were collected in the first three weeks of November 2023. The demographic characteristics of participants demonstrated that most participants identified as female (71.9%) and resided in North America (65.1%) (Table 2).

Among participants who self-identified as having EPI (*n* = 155), the gender distribution was also predominantly female (80.0%) and followed a similar geographic distribution and skewed slightly towards older age groups. Only five participants (3% of the 155 who self-reported having EPI) did not yet have a confirmed diagnosis. Further, of those with self-reported EPI, 77% reported their fecal elastase results, with a mean elastase of 103 μg/g (≤200 μg/g commonly considered indicative of EPI). In the subgroup without EPI (*n* = 169), most (64.5%) participants were female and had a higher representation from North America (57.4%), with age more evenly distributed.

Statistical analyses revealed a significant difference in EPI/PEI-SS total scores based on gender, with females reporting higher scores than males across all participants (*p* < 0.001), including within the EPI group. Age had no effect overall or within the EPI group. Many people with and without EPI also had other conditions that they self-reported (Table 3), but most conditions had no (type 1 or type 2 diabetes, chronic pancreatitis, celiac disease, GERD) or little (7–17% impact) additional impact (IBS, lactose or other food intolerances, gastroparesis) on EPI/PEI-SS total scores.

**Figure 1:**
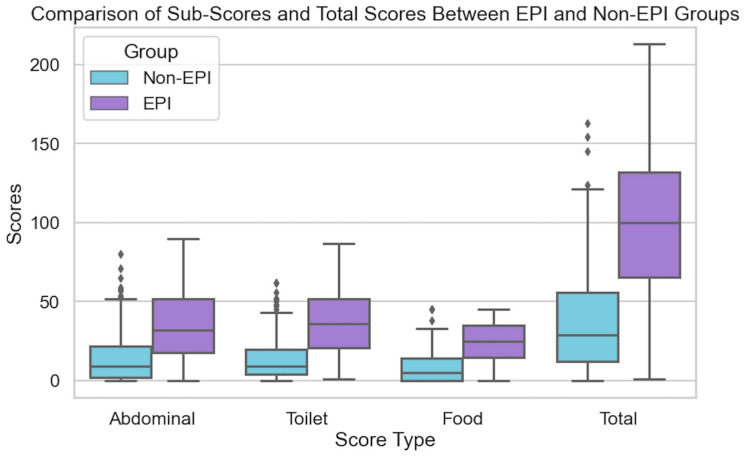
Comparison of sub-scores and total scores from EPI/PEI-SS in individuals with and without EPI.

**Figure 2:**
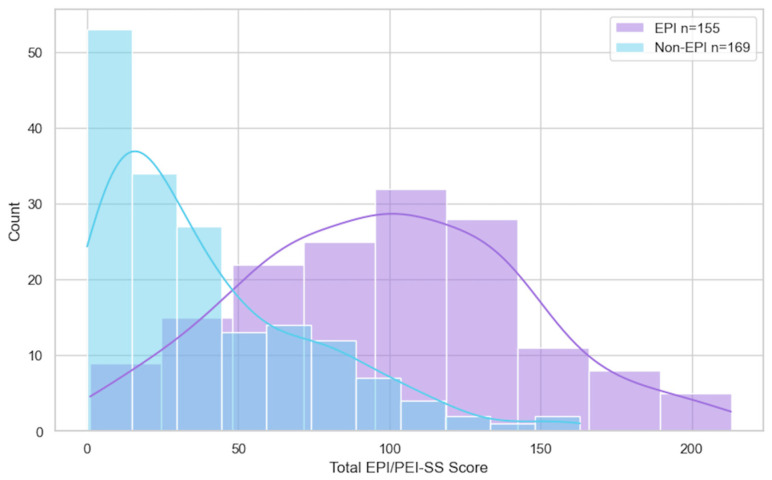
Total score distribution from EPI/PEI-SS in those with and without EPI.

There was a marked difference in symptom experience between the groups with and without EPI regarding each sub-score and the overall total score (max possible 225). The mean total score of those with EPI was 98.11 (min 1, max 213), in contrast to a mean total score of 38.86 for those without EPI (min 0, max 163). Sub-scores between the EPI and non-EPI groups and total score revealed the EPI group had statistically higher scores in all domains compared with the non-EPI cohort (Figure 1), and the distribution total score and count by group is also visualized (Figure 2).

The Mann–Whitney U test showed that all sub-scores and the total score were statistically significant (*p* < 0.001) in the differences between groups. Cohen’s d was evaluated (1.475) and determined to have a large (>0.8) effect size. Further, Cronbach’s alpha was assessed and determined to be “good” for all three sub-score categories (abdominal, 0.88; toilet, 0.83; food, 0.88) indicating high internal consistency within sub-groups, likely reliable scales, and good construct validity for these groupings of symptoms.

Overall, the area under the curve was 0.85 and with a score cutoff of 59 (out of possible 225), sensitivity was 0.81, and specificity was 0.75 in this population from the real-world study.

**Figure 3:**
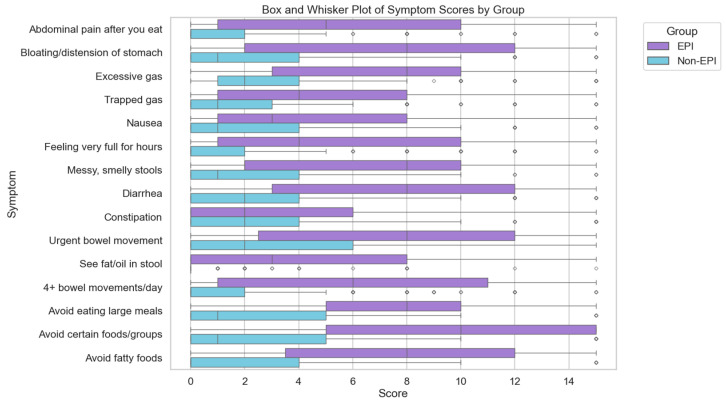
Individual symptom score distribution from EPI/PEI-SS in those with and without EPI.

Among people with self-reported EPI, individuals reported a mean of 12.39 symptoms with an average frequency score of 3.02 (once a week) and average severity score of 1.73 (between “slightly annoying” and “bothersome, and I wish I didn’t experience it”). Those without EPI reported not only four fewer symptoms, but each had nearly half the frequency and severity score, and the average individual symptom score was well under half that of those with EPI (mean symptoms, 8.15; mean frequency, 1.55; mean severity, 0.91). The symptom distributions are visualized in Figure 3.

**Figure 4:**
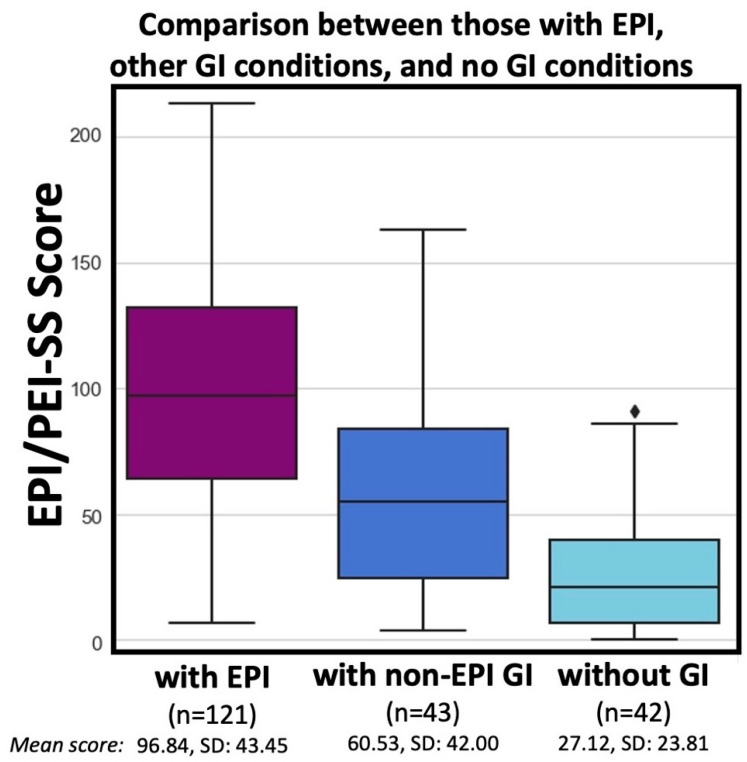
Total score distribution from EPI/PEI-SS in those with EPI compared to those with and without other gastrointestinal conditions.

Various GI-related conditions may have overlapping symptom profiles with EPI. To evaluate for the ability of the EPI/PEI-SS to distinguish between EPI and other non-EPI GI conditions, the group without EPI (*n* = 169, mean total score of 38.86) was broken down into those with (*n* = 72) and without other GI conditions (*n* = 97). The mean total score for the non-EPI with GI condition group was 55.94, in contrast to the mean non-EPI without GI score of 26.19. Paired *t*-tests and multiple other non-parametric tests (Shapiro–Wilk, Kruskal–Wallis, Mann–Whitney U with Bonferroni correction) confirm statistical significance (*p* < 0.001 for all group comparisons) when these two non-EPI groups with and without other GI conditions are compared with the EPI group. Non-EPI individuals with other GI conditions exhibit a substantially lower (statistically significant) symptom burden, less than half that than those diagnosed with EPI (Figure 4).

## 4. Discussion

This study developed and piloted a patient-designed symptom score tool (EPI/PEI-SS) for identifying possible cases of EPI, providing a novel method for distinguishing symptom frequency from self-reported symptom severity. The tool demonstrates good construct validity and internal consistency for diagnosing EPI, potentially helping patients with EPI access treatment they need to improve their quality of life. Our preliminary research findings suggest the EPI/PEI-SS can distinguish EPI symptoms from those caused by other gastrointestinal conditions, in a real-world population. Those who self-reported EPI had a higher gastrointestinal symptom burden than the general population, including people with other gastrointestinal conditions. People with EPI also reported more symptoms with increased frequency and severity than those without EPI, resulting in a significantly higher total mean score on the EPI/PEI-SS. Accordingly, the EPI/PEI-SS shows promise as a tool for helping clinicians screen appropriate patients for EPI, particularly by distinguishing these symptoms from other gastrointestinal conditions with overlapping symptom profiles.

While growing evidence indicates best-practice approaches for developing screening or diagnostic tools are those led by people with lived experience, few such tools exist. Other patient-reported instruments, such as one developed to help diagnose long COVID, were also found to be valid and reliable [27]. Previously developed tools [16] for EPI were designed primarily for healthcare providers and amalgamate symptom frequency and severity. In addition, these tools often were validated only in sub-populations such as those with cystic fibrosis or chronic pancreatitis [16], excluding the predominant cohorts of people living with EPI, such as those with diabetes or celiac disease [21]. Our study utilized a diverse population of people diagnosed with EPI, not small sub-groups of co-conditions, likely the result of the involvement of people with lived experience.

This real-world study included a comparison group of people who did not report having EPI. This is critical as previous population studies have illustrated that the average person, even without medical conditions, experiences gastrointestinal symptoms regularly [28,29]. In our study, the EPI/PEI-SS scores for the non-EPI population showed a mean of 8 symptoms (out of possible 15), with a mean frequency of less than once a month, indicating that our real-world ‘general’ population (without identified EPI) is representative of previously studied ‘general’ populations [29]. Thus, the comparison of substantially higher EPI/PEI-SS score among individuals with EPI illustrates a practically, and statistically, significant symptom burden distinguishable from non-pathological gastrointestinal symptoms. We also observed a statistically significant difference in EPI/PEI-SS scores based on gender, with females reporting higher scores in all groups. This finding matches previous literature which suggests higher prevalence and impact of gastrointestinal conditions in females [30]. A higher score on the EPI/PEI-SS may signal the need for the patient to undergo follow-up testing for EPI, especially if symptoms align with those of pancreatic insufficiency [31,32].

Future studies of the EPI/PEI-SS, combined with fecal elastase testing, are planned and being conducted in several countries in the general population, as well as in sub-populations such as people with diabetes and people with pancreatic cancer, among others. In the future, this tool could be used online, including evaluating showing the score [33], as well as offline with traditional paper surveys with or without score presentation. These studies may further help evaluate the EPI/PEI-SS and could allow trials of PERT based on EPI/PEI-SS scores as an alternative to burdensome fecal testing, ultimately increasing access to PERT for people with EPI. Further, the EPI/PEI-SS could be used to track PERT efficacy over time. A reduction in scores closer to non-EPI mean scores could indicate successful treatment with PERT, and an absence of score reduction may suggest further diagnostic consideration [22]. Future studies could include additional imaging and diagnostic methods to assess for undiagnosed cases of other conditions, especially gastrointestinal conditions where there may be overlapping symptom profiles.

Limitations of this study include that the design was primarily exploratory and descriptive to assess hypothesized differences in EPI/PEI-SS results between EPI and non-EPI participants and it was not powered for a pre-specified outcome (such as variations within the EPI group itself). The tool will also need to be further tested to associate outcomes in terms of fecal elastase results and/or other diagnostic methods to determine whether the EPI/PEI-SS could be used to proceed to a trial of PERT. This should also include a more diverse set of participants with multiple ethnicities, as although there was geographic diversity (Table 2), ethnicities and socioeconomic status were not captured in the survey. The majority of participants were also female, and although score differences between females and males were statistically significant this should be evaluated in subsequent studies with increased male participation. Additionally, this study was conducted online and via various social media platforms. This may raise the issue of diagnostic accuracy among some clinicians, although evidence suggests that self-reported outcomes (specifically including gastrointestinal symptoms) from online/digital surveys overlap those of traditional, in-person clinical studies [28]. In this study, although self-reporting EPI status, most participants did have a fecal elastase level aligned with EPI diagnosis and also a prescription for pancreatic enzyme replacement therapy from a provider, indicating formal existing diagnosis (97%) to match self-reported status. As it is possible our participants did not fully represent people with EPI, the study could be repeated using clinically-identified populations of EPI to further validate the EPI/PEI-SS. Sub-group analyses were performed to confirm that despite the over-representation of people with type 1 diabetes in the non-EPI group, there were no practical nor statistically significant differences influencing the symptom scores between the diabetes and non-diabetes groups [26].

## 5. Conclusions

This is the first symptom score directly developed by people living with EPI, measuring what matters most to them, and one which enables individuals to quantify which symptoms are the most frequent and/or the most burdensome (severe) to their quality of life. Our findings provide pilot evidence for the EPI/PEI-SS in detecting the broader populations of people living with EPI, without limiting focus to specific sub-populations, and could be used in future clinical studies. Ultimately, the use of the EPI/PEI-SS may eventually enhance therapeutic outcomes and contribute to increasing the ability to successfully diagnose EPI and improving the quality of life for those living with EPI.

## Figures and Tables

**Table 1:** The individual symptoms within each of three groups of EPI-related symptoms, and the total possible score for each sub-group ^1^.

Group	Individual Symptom	Score
Abdominal-Related Symptoms	Abdominal pain after eating	90
	Bloating/distension of stomach	
	Gas	
	Trapped gas	
	Nausea	
	Feel very full hours after meal (e.g., did not eat lunch because still full from breakfast)	
Toilet-Related Symptoms	Messy, smelly stools that stick to the side of the toilet bowl	90
	Diarrhea	
	Constipation	
	Urgent bowel movement (rush to the toilet)	
	See fat or oil in stool or on toilet paper	
	4 or more bowel movements per day	
Food-Related Symptoms	I avoid eating large meals	45
	I avoid certain foods or food groups (excluding food allergies or celiac)	
	I avoid fatty foods	
	Total Score Possible:	225

^1^ For frequency, scores reflect never (0); at least once, but not regularly (1); once a month (2); once a week (3); a few times a week (4); or most days of the week (5). For severity, scores reflect not having the symptom (0); slightly annoying (1); bothersome and I wish I didn’t experience it (2); and very bothersome and impacts my quality of life (3). Each symptom score is created by multiplying frequency by severity for a possible item score of 0–15, then added up by sub-group and to the total EPI/PEI-SS score possible of 0–225.

**Table 2:** Demographic characteristics of participants, with and without EPI.

		With EPI Count (Percentage)	Without EPI Count (Percentage)	Overall Count (Percentage)
**Gender**				
	Female	124 (80.0%)	109 (64.5%)	233 (71.9%)
	Male	30 (19.4%)	57 (33.7%)	87 (26.9%)
	Non-binary	1 (0.6%)	2 (1.2%)	3 (0.9%)
	Prefer not to say	0 (0.0%)	1 (0.6%)	1 (0.3%)
**Location**				
	North America	114 (73.5%)	97 (57.4%)	211 (65.1%)
	Europe (including UK)	35 (22.6%)	53 (31.4%)	88 (27.2%)
	Australia	2 (1.3%)	9 (5.3%)	11 (3.4%)
	Asia	2 (1.3%)	2 (1.2%)	4 (1.2%)
	New Zealand	2 (1.3%)	3 (1.8%)	5 (1.5%)
	South America	0 (0.0%)	3 (1.8%)	3 (0.9%)
	Other	0 (0.0%)	2 (1.2%)	2 (0.6%)
**Age Group**				
	<18	1 (0.6%)	3 (1.8%)	4 (1.2%)
	18–29	7 (4.5%)	13 (7.7%)	20 (6.2%)
	30–39	22 (14.2%)	41 (24.3%)	63 (19.4%)
	40–49	26 (16.8%)	52 (30.8%)	78 (24.1%)
	50–59	46 (29.7%)	36 (21.3%)	82 (25.3%)
	60–69	38 (24.5%)	21 (12.4%)	59 (18.2%)
	70–79	14 (9.0%)	2 (1.2%)	16 (4.9%)
	80 or older	1 (0.6%)	1 (0.6%)	2 (0.6%)
	**TOTAL**	155	169	324

**Table 3:** Other conditions and frequency among those with and without EPI.

Condition	Individuals with EPI Count (Percentage)	Individuals Without EPI Count (Percentage)
Type 1 diabetes	14 (6%)	78 (41%)
IBS	43 (18%)	24 (13%)
Lactose intolerance	26 (11%)	27 (14%)
GERD	32 (14%)	18 (9%)
Other food intolerances	28 (12%)	17 (9%)
Chronic pancreatitis	21 (9%)	1 (1%)
Gastroparesis (delayed gastric emptying)	17 (7%)	7 (4%)
Celiac disease	9 (4%)	7 (4%)
Acute pancreatitis	11 (5%)	4 (2%)
Type 2 diabetes	20 (9%)	6 (3%)
Pancreatic surgery	4 (2%)	0 (0%)
Endometriosis	1 (0%)	1 (1%)
Lupus	1 (0%)	2 (1%)
Pancreatic cancer	2 (1%)	0 (0%)
Hashimoto’s	2 (1%)	0 (0%)
Sjogren’s	2 (1%)	0 (0%)
**TOTAL**	**233**	**192**

## Data Availability

Raw data (anonymized) is available upon request to the corresponding author.

## Abbreviations

The following abbreviations are used in this manuscript:

ANOVA	Analysis of variance
CP	Chronic pancreatitis
CF	Cystic fibrosis
EPI/PEI	Exocrine pancreatic insufficiency/pancreatic exocrine insufficiency
EPI/PEI-SS	Exocrine Pancreatic Insufficiency Symptom Score
GERD	Gastroesophageal reflux disease
GI	Gastroenterology
HSD	Honestly significant difference
IBS	Irritable bowel syndrome
PERT	Pancreatic enzyme replacement therapy
